# A novel, reusable, realistic neurosurgical training simulator for cerebrovascular bypass surgery: Iatrotek^®^ bypass simulator validation study and literature review

**DOI:** 10.3389/fsurg.2023.1048083

**Published:** 2023-02-09

**Authors:** Marcello D’Andrea, Antonio Musio, Roberto Colasanti, Lorenzo Mongardi, Dalila Fuschillo, Giorgio Lofrese, Luigino Tosatto

**Affiliations:** ^1^ Department of Neurosurgery, Maurizio Bufalini Hospital, Cesena, Italy; ^2^ Department of Ferrara – Neurosurgery, Sant ‘Anna University Hospital, Ferrara, Italy; ^3^ Department of Neurosurgery, Padua University Hospital, Padua, Italy

**Keywords:** vascular neurosurgery, bypass, microanastomosis, bypass simulator, neurosurgical training

## Abstract

**Background:**

Microanastomosis is a challenging technique requiring continuous training to be mastered. Several models have been proposed, but few effectively reflect a real bypass surgery; even fewer are reusable, most are not easily accessible, and the setting is often quite long. We aim to validate a simplified, ready-to-use, reusable, ergonomic bypass simulator.

**Methods:**

Twelve novice and two expert neurosurgeons completed eight End-to-End (EE), eight End-to-Side (ES), and eight Side-to-Side (SS) microanastomoses using 2-mm synthetic vessels. Data on time to perform bypass (TPB), number of sutures and time required to stop potential leaks were collected. After the last training, participants completed a Likert Like Survey for bypass simulator evaluation. Each participant was assessed using the Northwestern Objective Microanastomosis Assessment Tool (NOMAT).

**Results:**

When comparing the first and last attempts, an improvement of the mean TPB was registered in both groups for the three types of microanastomosis. The improvement was always statistically significant in the novice group, while in the expert group, it was only significant for ES bypass. The NOMAT score improved in both groups, displaying statistical significance in the novices for EE bypass. The mean number of leakages, and the relative time for their resolution, also tended to progressively reduce in both groups by increasing the attempts. The Likert score expressed by the experts was slightly higher (25 vs. 24.58 by the novices).

**Conclusions:**

Our proposed bypass training model may represent a simplified, ready-to-use, reusable, ergonomic, and efficient system to improve eye-hand coordination and dexterity in performing microanastomoses.

## Introduction

The introduction of endovascular techniques has progressively reduced the number of open surgeries for cerebrovascular diseases. Consequently, neurosurgical residents and young trainees have fewer opportunities to see, learn and practice cerebrovascular procedures. Nonetheless, bypass surgery still plays a crucial role in treating complex aneurysms, moyamoya disease, and in restoring adequate cerebral circulation when dealing with difficult intracranial tumors or other occlusive vascular lesions (1–8).

Microvascular anastomosis is one of the most challenging neurosurgical technique because of the small diameter of the vessels (less than 2 mm in most of the cases) as well as the depth and the narrowness of the surgical field. Hence, microvascular anastomosis techniques require dedicated and effective training to be mastered. Several training models, such as chicken wings, rats, placental vessels, human cadavers, and plastic tubing have been suggested for improving and refining microsurgical skills (2, 9–15). In the last years, virtual reality models and web-based simulators have also proved to be very powerful tools in enhancing trainees' learning experience at various levels (1, 16–22).

However, the proposed training models do not often adequately reflect the realism of an actual cerebrovascular bypass surgery and/or are not easily accessible. Here, we describe a simplified, ready-to-use, reusable, ergonomic simulator for training microvascular anastomosis techniques (1, 16–22).

## Materials and methods

### Iatrotek^®^ bypass simulator: technical device description

**Figure 1:**
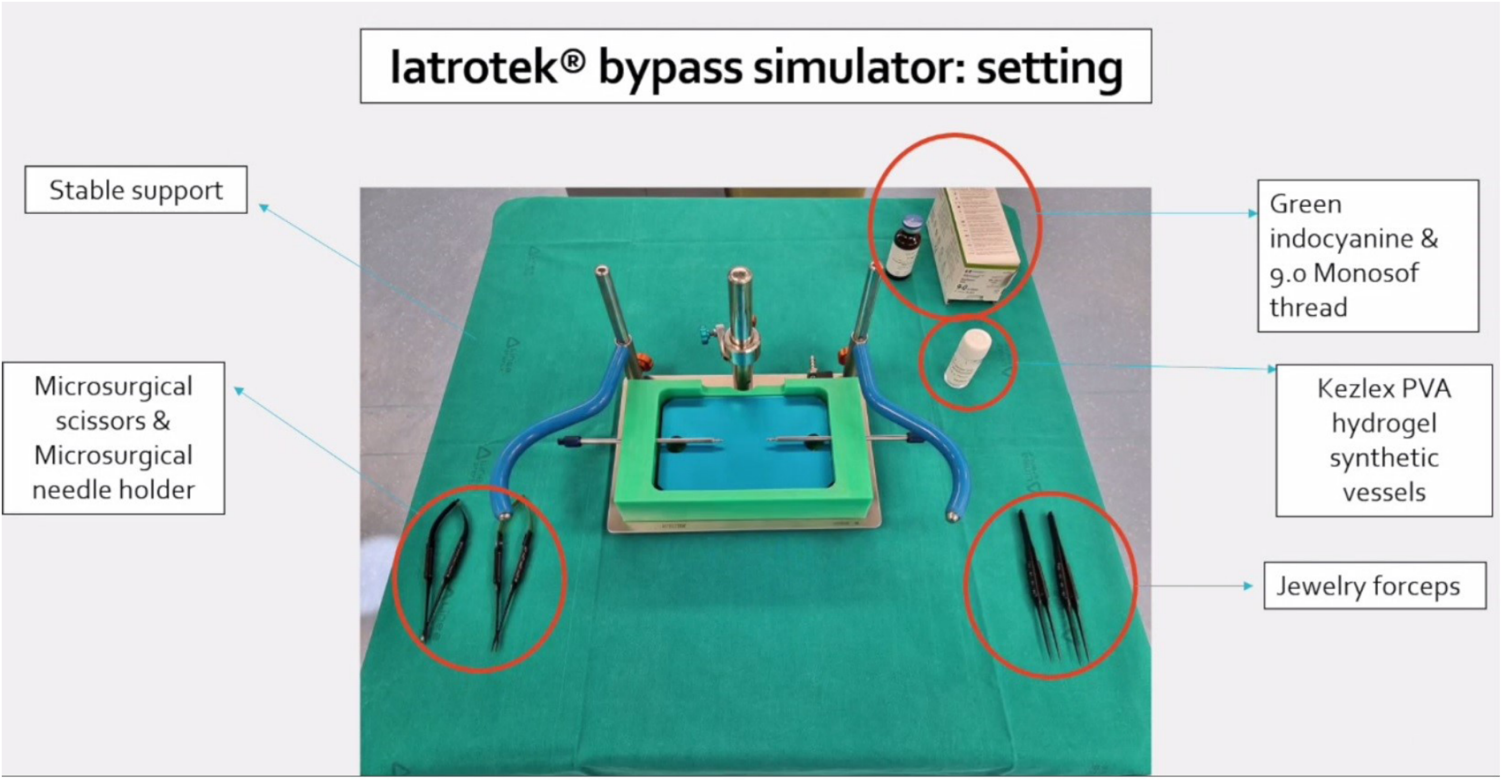
Setting of Iatrotek bypass simulator. The bypass simulator is placed on a stable support. Microsurgical scissors and needle holder are shown and tagged, together with Jewelry forceps, Kezlex Polyvinyl alcohol (PVA) hydrogel synthetic vessels, Indocyanine green and 9.0 Monosof thread.

**Figure 2:**
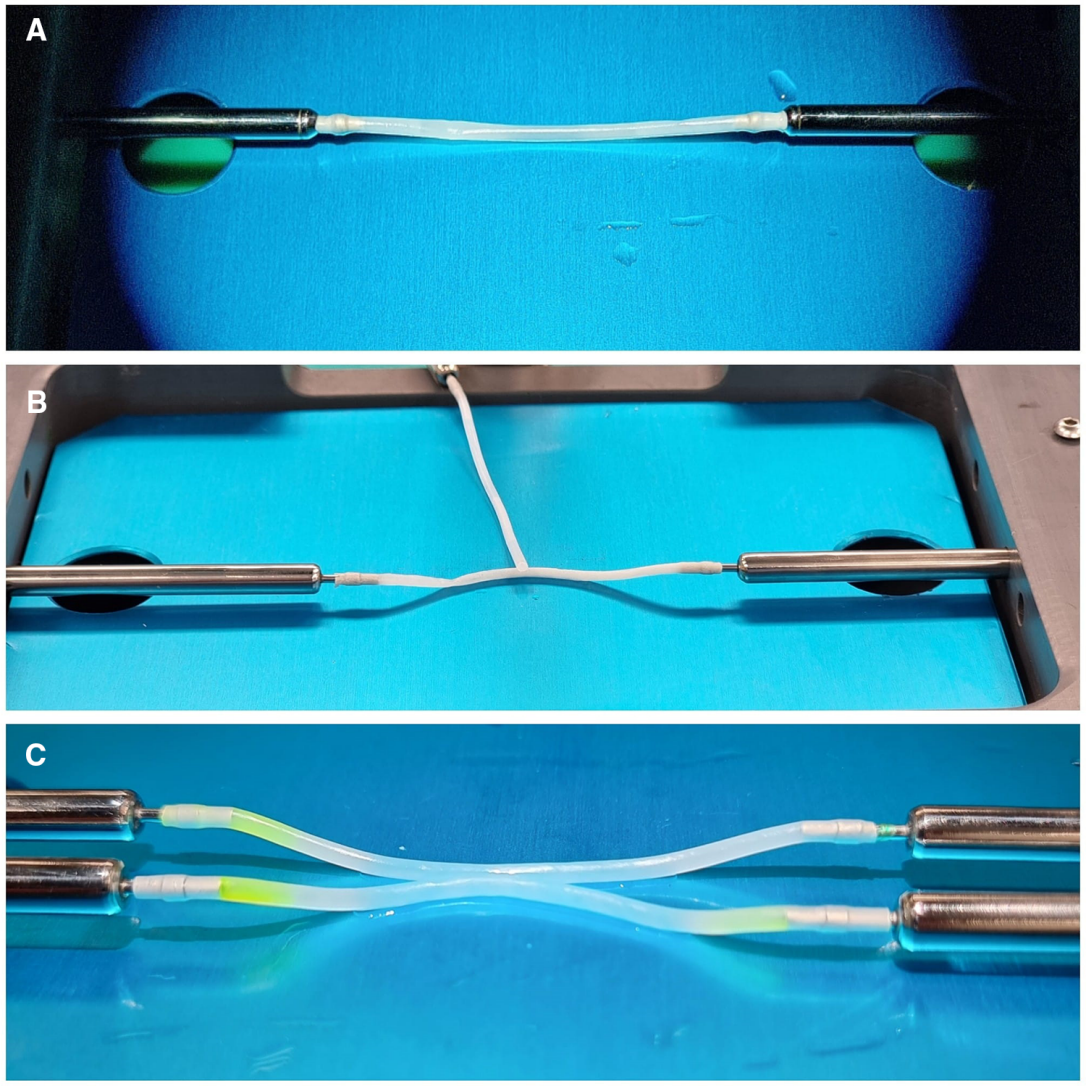
Details of Kezlez PVA hydrogel vessels. Setting for EE, ES and SS microanastomosis is shown in picture (**A–C**) respectively.

The bypass simulator consists of a rectangular main structure (200 mm × 300 mm) in alloy EN-AW 6082/T6 with anodic oxidation treatment, stainless steel (X5CrNi 18/10), polymethylacrylate and polyvinyl chloride (C2H3Cl)n, with a maximum high of 250 mm. The platform's weight is 0.3 Kg. Several optional accessories are available: a mechanism to modify the depth of the working field; a bypass extender; 1, 2, and 3 mm holders for artificial vessels; set of short and long microsurgical instruments; indocyanine green; set of long needles for side-to-side anastomosis; and Kezlex Polyvinyl alcohol (PVA) hydrogel synthetic vessels with various diameters (1 mm, 2 mm, 3 mm for a length of 70 mm) (Figure 1). The PVA hydrogel vessels are qualitatively similar to the human donor and recipient arteries that are usually involved in bypass surgeries because their production is based on three-dimensional computed tomography/magnetic resonance imaging scanning data. Furthermore, several studies have confirmed that PVA hydrogel vessels represent a good biomaterial for practicing microsurgical anastomosis as they are similar to real arteries for mechanical properties (e.g., surface friction and elasticity), as well as for their transparency and for a comparable staining with blue dye (Figure 2) (23).

### Iatrotek^®^ bypass simulator: setting

The training platform has to be placed on a stable support (I).

A mechanism permits to adjust the depth of the working field in accord with the desired degree of difficulty. In addition, a diaphragm is used to set the width of the working field (II).

Interchangeable approximators are inserted in the corresponding holes in relation to the size of the vessels that will be fixed to their nozzles. The length and tension of the vessels may be adjusted by moving the approximators (III).

The approximators are hollow and connected with a fluid pump. This allows a continuous or an “on demand” irrigation of the vessels (simulating the blood flow), as well as the use of the indocyanine green to check the tightness of the anastomosis (IV).

**Figure 3:**
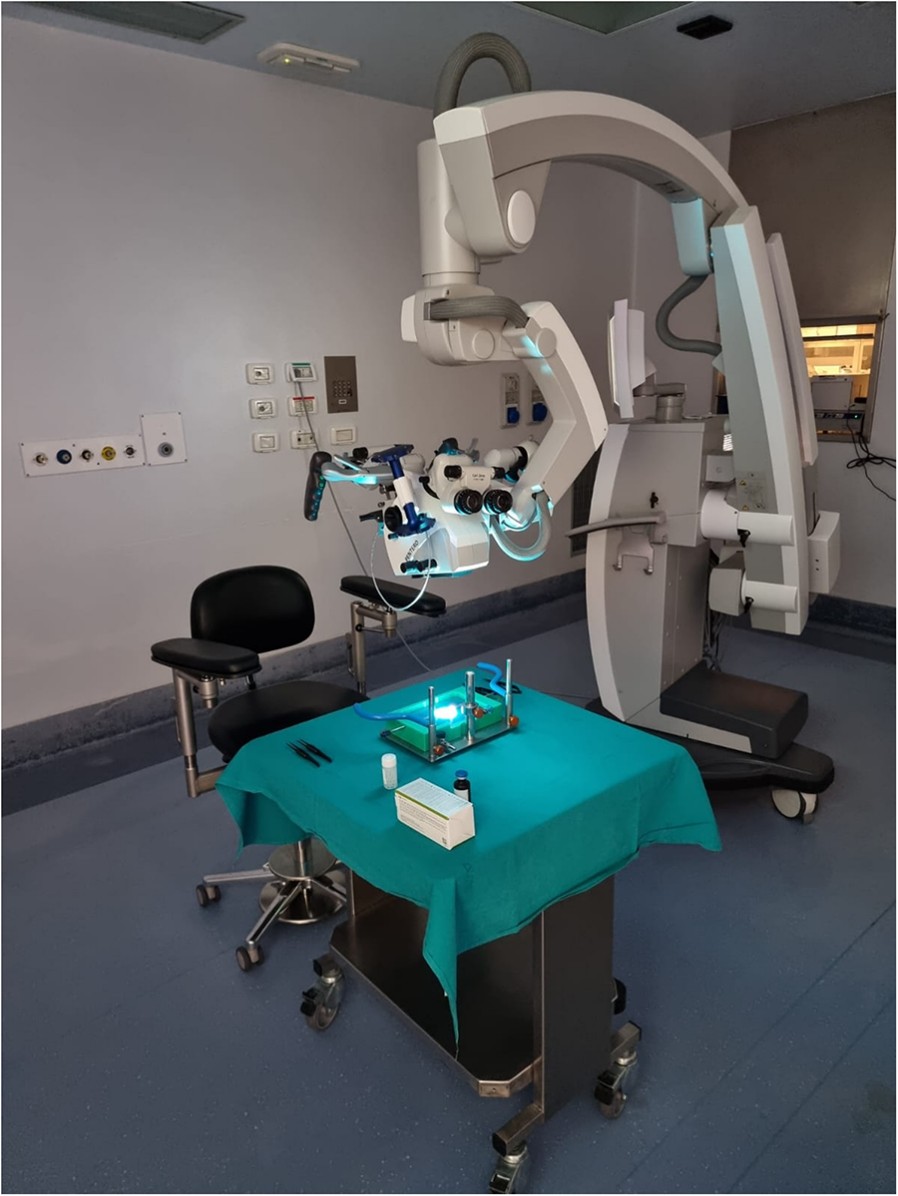
Workstation. The training platform has to be placed on a stable support. Workstation place is very versatile: it could be set in a laboratory, on study room desk or, as in this case, in the surgery room.

The setting procedure takes usually less than 5 min before the training could start (Supplementary Video S1 and Figure 3).

### Participants

Ten neurosurgical residents and two postdoctoral research fellows (“novice group”) were enrolled in the study together with two expert vascular neurosurgeons (“expert group”). In 80 days, each trainee completed eight End to End (EE), eight End to Side (ES) and eight Side to Side (SS) microanastomoses using 9.0 Monosof™ interrupted sutures and 2-mm synthetic vessels. All the participants performed each trial (consisting of the 3 different microanastomoses) every ten days. Data on number of knots (NOK), time to perform bypass (TPB), number of sutures and time required to stop potential leaks after bypass execution were collected. Indocyanine green and/or fluoresceine were used for all the procedures showing leaks when present.

**Figure 4:**
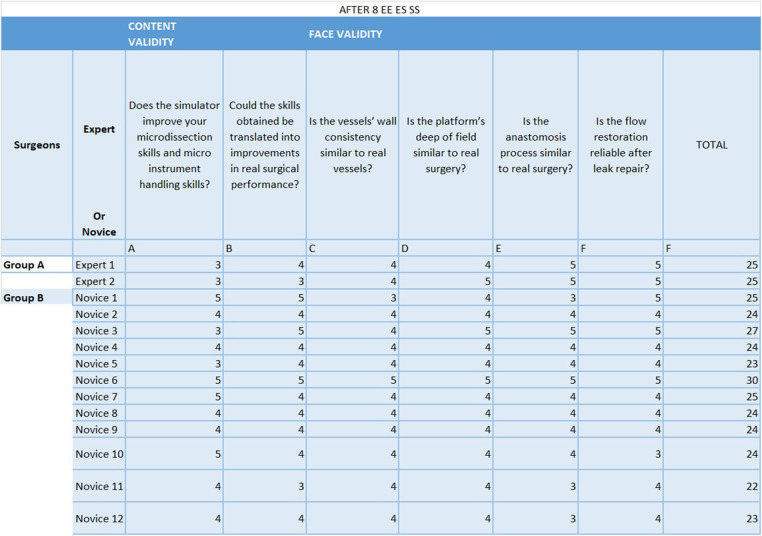
6-question Likert Like Survey. Each question was answered, after the 8th attempt by each of the partecipants, on a scale from 1 to 5: 5- exactly like; 4- very similar; 3– similar; 2– little similarity; 1– not similar, with the statements provided. The first four questions assessed face validity: the participants judged the degree of actual task replication and difficulty when compared to real surgery. The other questions appraised content validity, i.e. the potential of the training platform to upgrade their microdissection and microinstrument handling skills and the likelihood that this could determine improvements in their real surgical performance.

After completing the last training, participants completed a 6-question Likert Like Survey (Figure 4) in order to evaluate the bypass simulator (1). Each question was answered on a scale from 1 to 5: 5– exactly like; 4– very similar; 3– similar; 2– little similarity; 1– not similar, with the statements provided. The first four questions assessed face validity: the participants judged the degree of actual task replication and difficulty when compared to real surgery. The other questions appraised content validity, i.e., the potential of the training platform to upgrade their microdissection and microinstrument handling skills and the likelihood that this could determine improvements in their real surgical performance (1).

The overall performance of each participant was evaluated using the Northwestern Objective Microanastomosis Assessment Tool (NOMAT) (24). Two cameras recorded all the training procedures. One expert vascular neurosurgeon (MDA) blindly graded the performance of the participants, which were divided into a “novice group” and an “expert group” as described above.

### Statistical analysis

A statistical analysis was performed using SPSS software (version 20; SPSS Inc., Chicago, IL, USA) in order to detect any difference between the two groups (“novice group” vs. “expert group”) at the first and at the last attempt with the three different types of microanastomosis. In addition, we searched for any difference in the overall performance in the two groups, comparing the overall results of the participants at their first and last attempts, in order to evaluate the potential improvement due to the use of the training platform.

The statistical analysis of data was carried out by the Pearson chi-square test for discrete variables and the *t*-test for continuous ones. The statistical significance was set at *p* < 0.05.

## Results

A total of 336 bypasses were performed (24 for each trainee, 8EE, 8ES, 8SS). Detailed results are displayed in Tables 1–3.

**Table 1:** Results of the NOMAT score and objective task evaluation of the 2 mm vessels end-to-end bypass in each group at 1st and 8th attempts.

2 mm vessels End-to-End bypass	Experienced (*n* = 2) 1st attempt	Experienced (*n* = 2) 8th attempt	*p*-value Experienced 1st vs. Experienced 8th attempt	Novice (*n* = 12) 1st attempt	Novice (*n* = 12) 8th attempt	*p*-value Novice 1st vs. Novice 8th attempt	*p*-value Experienced 1st vs. Novice 1st attempt	*p*-value Experienced 8th vs. Novice 8th attempt
Mean Number of Knots (range)	7.5 (7–8)	7 (7–7)^a^	0.423	8.17 (6–9)	7.75 (6–9)	0.338	0.437	0.309
Mean Time to perform bypass in min (range)	19.2 (16–22.4)	17.3 (17–17.6)	0.614	37.25 (30–42)	21.25 (16–28)	***<0***.***0005***	***<0***.***0005***	0.202
Number of Leakages after bypass execution (%)	2 (100%)	1 (50%)	0.248	9 (75%)	6 (50%)	0.206	0.425	1.000
Mean Number of sutures needed to stop leak (range)	2 (2–2)^a^	2	Not applicable	1.78 (1–3)	1.86 (1–3)	0.842	0.726	0.853
Mean Time to stop leak in min (range)	7.50 (6–9)	9	0.667	6.44 (4–9)	6.29 (4–9)	0.854	0.453	0.187
Mean NOMAT^b^ score (range)	44 (40–48)	45.5 (45–46)	0.746	20.5 (16–30)	33.75 (22–49)	***<0***.***0005***	***<0***.***0005***	0.150

^a^ Two experienced participants scored the same in both trials.

^b^ Score ranges for NOMAT (0–70).

In italic and bold the variables with *p* < 0.05.

**Table 2:** Results of the NOMAT score and objective task evaluation of the 2 mm vessels end-to-Side bypass in each group at 1st and 8th attempts.

2 mm vessels End-to- Side bypass	Experienced (*n* = 2) 1st attempt	Experienced (*n* = 2) 8th attempt	*p*-value Experienced 1st vs. Experienced 8th attempt	Novice (*n* = 12) 1st attempt	Novice (*n* = 12) 8th attempt	*p*-value Novice 1st vs. Novice 8th attempt	*p*-value Experienced 1st vs. Novice 1st attempt	*p*-value Experienced 8th vs. Novice 8th attempt
Mean Number of Knots (range)	7 (7–7)^a^	8 (8–8)^a^	Not applicable	8.17 (7–9)	7.83 (6–9)	0.368	***0***.***046***	0.829
Mean Time to perform bypass in min (range)	20.20 (20–20.4)	18.70 (18.7–18.7)^a^	***0***.***017***	41.58(30–49)	28.83 (20–40)	***<0***.***0005***	***<0***.***0005***	***0***.***019***
Number of Leakages after bypass execution (%)	2 (100%)	1 (50%)	0.248	11 (91.7%)	8 (66.7%)	0.132	0.672	0.649
Mean Number of sutures needed to stop leak (range)	2 (2–2)^a^	2	Not applicable	1.64 (1–2)	1.63 (1–2)	0.962	0.347	0.516
Mean Time to stop leak in min (range)	7.5 (6–9)	9	0.667	6.82 (4–9)	5.75 (4–9)	0.194	0.643	0.078
Mean NOMAT^b^ score (range)	44.25 (43.5–45)	48 (47–49)	0.095	20.50 (16–32)	20.58 (12–24)	0.959	***<0***.***0005***	***<0***.***0005***

^a^ Two experienced participants scored the same in both trials.

^b^ Score ranges for NOMAT (0–70).

In italic and bold the variables with *p* < 0.05.

**Table 3:** Results of the NOMAT score and objective task evaluation of the 2 mm vessels Side-to-Side bypass in each group at 1st and 8th attempts.

2 mm vessels Side-to-Side bypass	Experienced (*n* = 2) 1st attempt	Experienced (*n* = 2) 8th attempt	*p*-value Experienced 1st vs. Experienced 8th attempt	Novice (*n* = 12) 1st attempt	Novice (*n* = 12) 8th attempt	*p*-value Novice 1st vs. Novice 8th attempt	*p*-value Experienced 1st vs. Novice 1st attempt	*p*-value Experienced 8th vs. Novice 8th attempt
Mean Number of Knots (range)	8.50 (8–9)	7.50 (7–8)	0.293	8.33 (7–9)	8.08 (6–9)	0.523	0.807	0.449
Mean Time to perform bypass in min (range)	23.00 (20–26)	22.4 (17.3–27.5)	0.928	40.67 (30–49)	28.83 (20–40)	***<0***.***0005***	***0***.***001***	0.139
Number of Leakages after bypass execution (%)	2 (100%)	2 (100%)	Not applicable	12 (100%)	11 (91.7%)	0.307	Not applicable	0.672
Mean Number of sutures needed to stop leak (range)	2(2–2)^a^	2 (2–2)^a^	Not applicable	1.9 (1–3)	1.82 (1–3)	0.783	0.857	0.689
Mean Time to stop leak in min (range)	7.5 (6–9)	7.5 (6–9)	1.00	7.2 (5–9)	6.73 (5–9)	0.494	0.822	0.532
Mean NOMAT^b^ score (range)	39.25 (38–40.5)	43 (43–43^a^)	0.095	33.75 (22–49)	33.75 (22–49)	1.00	0.486	0.250

^a^ Two experienced participants scored the same in both trials.

^b^ Score ranges for NOMAT (0–70).

In italic and bold the variables with *p* < 0.05.

Novice group average TPB (minutes) at the first vs. at the eighth attempt was 37.25 vs. 21.25 min for EE (*p* < 0.0005), 41.58 vs. 28.83 min for ES (*p* < 0.0005), 40.67 vs. 28.83 min for SS (*p* < 0.0005).

The mean NOMAT score for the expert group at the first vs. at the eighth attempt was 44 vs. 45.5 for EE (*p* = 0.746), 44.25 vs. 48 for ES (*p* = 0.095), 39.25 vs. 43 for SS (*p* = 0.095). The mean NOMAT score for the novice group at the first vs. at the eighth attempt was 20.5 vs. 33.75 for EE (*p* < 0.0005), 20.50 vs. 20.58 for ES (*p* = 0.959), 33.75 vs. 33.75 for SS (*p* = 1.00).

The mean Likert score after the last attempt of microanastomosis was 25 for the expert group and 24.58 for the novice group out of a maximum score of 30 (Figure 3).

## Discussion

Residents and young surgeons require a continuous training process in order to develop eye-hand coordination and dexterity in performing microvascular anastomosis techniques. However, the proposed training models do not often adequately reflect the realism of an actual cerebrovascular bypass surgery and/or are not easily accessible (Table 4).

**Table 4:** Summary of the advantages and disadvantages of available bypass training models for practicing microvascular anastomosis.

Bypass training model	Advantages	Disadvantages
Human cadaveric head/brain	Anatomically the most realistic and the closest model to live surgery	Scarce opportunities for working with this model on individual basis: educational programs like cadaver dissection courses are required; expensive; infection risk
Swine artery; Chicken wing model	Size and feel closely resemble those of human vessels; inexpensive and easily obtainable at the grocery store or from a farm	Not ready-made for practicing: the removal of the fat and connective tissue around the vessels is required before starting microvascular anastomosis; vessels of the desired size and length may not be always harvested; potential infection risk
Rat	Pulsatile blood flow; natural viscosity; size and feel very similar to those of human vessels; real potential for thrombosis	Require dedicated laboratories; high costs; ethical issues; need careful and adequate dissection before starting the microanastomosis training; vessels may not have the desiderable size and/or length; infection risk
Human placenta	Placental vessels offer a great similarity to main brain vessels; high face, content, and construct validities; may provide material for dozens of microsurgical anastomoses in a single session	A specific distribution route has to be arranged with the Obstetrics and Gynaecology unit (hence, may not be suitable for individual use); storage issues; not ready-made for practicing: placenta vessels have to be adequately prepared before microanastomosis training; infection risk
Grapefruit model	Grapefruits and human brains have similar dimensions; inexpensive, realistic, reusable	Specifically designed for training only the distal anterior cerebral artery side-to-side bypass; its assembly is not so immediate
Silicone tube	Reusable, easy set-up, long duration, no infection risk	Different feel compared to real vessels (hard and inflexible without moisture)
PVA hydrogel tube (KEZLEX)	Qualitatively similar surface friction, transparency, and elasticity to real human vessels; user-friendly (as described for the silicone tube); no infection risk	Easily dries (needs to be kept moist); short length (6–8 cm); more expensive than silicone tubes
IATROTEK MODEL with KEZLEX	Ready-to-use, easy to set; reusable, realistic, ergonomic simulator where PVA hydrogel tubes (see above) are easily placed and used for anastomosis. Vessels can be continuously injected with water and/or Indocyanine green to detect any leak. The depth of the working field may be adjusted in accord with the desired degree of difficulty	Does not allow vessels dissection, sulcus dissection; not as high fidelity as human placenta or animal models; more expensive than other models above described

In the past years live animals (in particular rats) were the most adopted training model. The main advantages of this model are obviously represented by the pulsatile blood flow and natural viscosity. Furthermore, the size and the feel of animal arteries resemble those of human vessels (1, 25). However, nowadays, increasingly strict regulations concerning animal welfare, appropriate shelter, economic and ethical issues, and surgical management have progressively imposed significant but necessary limitations of the use of live animal models. In addition, animals models have to be adequately prepared before starting a microvascular anastomosis training: a careful dissection of the surrounding fat and connective tissue is required to expose the vessels, and sometimes the vessels may not have the desirable size and/or length (1, 25).

For these reasons, other bypass training models have been evaluated in the last decades. Fresh human and bovine placentas proved to be excellent tissue models for bypass training, because their vessels are greatly similar to the cerebral ones, and a placenta may provide material for dozens of microsurgical anastomoses in a single session. Several placenta training models have been proposed and described in the medical literature (26–28). Oliveira et al. proposed a human placenta simulator, a high-fidelity and easily available simulator for training neurosurgeons in vascular microsurgery (17, 29). Belykh et al. demonstrated also that human and bovine placental vessels are convenient, anatomically relevant, and beneficial models for microneurosurgical training. Hence, the microanastomosis simulation using these models has high face, content, and construct validities (1). In 2020, Ferrarez et al. described a superficial temporal artery-middle cerebral artery bypass training simulator placing a human placenta in an artificial skull in which a fronto-temporal approach had been previously performed (18).

On the other hand, the use of placenta vessels has also several disadvantages: (1) scarce opportunities to work on these models on an individual basis (educational programs are often required); (2) a specific distribution route has to be arranged with the Obstetrics and Gynaecology unit; (3) storage issues; (4) placenta vessels, like animals' models, have to be adequately prepared before starting a microvascular anastomosis training.

Beyond live animals and placentas, several artificial models have been proposed and described in the medical literature. In 2011, Mori et al. proposed a simulation model for training posterior circulation revascularization: a three-dimensional skull model with artificial brain, where superior cerebellar and posterior inferior cerebellar arteries were made from artificial blood vessels and glued on the brain (30). In 2020, Cikla et al. described the “grapefruit training model” which may provide a realistic simulation of side-to-side distal anterior cerebral artery bypass procedure using a “dissected grapefruit” to simulate the interhemispheric fissure, chicken wing vessels or synthetic tubing for pericallosal arteries, and an aquarium pump to mimic circulation (31). The main disadvantage of this model is that its assembly is not so immediate (in particular if the vessels are harvested from chicken wings). In addition, the model is specifically designed for training only the distal anterior cerebral artery side-to-side bypass (31).

In consideration of the above, even if it is evident that several and different bypass training models have been proposed so far, few reflect the realism of an actual cerebrovascular bypass surgery, even fewer are reusable, most are not easily accessible, and the setting procedure before starting the bypass training is not so fast in almost all the models.

The setting procedure of the Iatrotek® bypass simulator is really quick, usually less than 5 min before the training could start. Our training model is reusable, and may be easily transported and used in any available place.

Obviously, the Kezlex PVA hydrogel synthetic vessels do not allow to train dissection skills, but they are more readily available, and easier to handle and store than live animals, placentas or artificial models. Then, the PVA hydrogel vessels are produced using three-dimensional computed tomography/magnetic resonance imaging scanning data, and their mechanical properties (e.g., surface friction and elasticity) and transparency are very similar to real arteries. In addition, the patency and the tightness of the bypass may be tested irrigating the vessels or using the indocyanine green.

Finally, when using the Iatrotek® bypass simulator, the degree of difficulty of the training may be easily and quickly adjusted modifying the depth and the width of the working field, as well as the position of the interchangeable approximators.

Our experience showed that the training with the Iatrotek® bypass simulator was able to determine a performance improvement in both the novice and expert groups. When comparing the results of the first and the last attempts, we registered an improvement in the mean TPB in both groups for all the three types of microanastomosis. The improvement was obviously more pronounced in the novice group, always reaching a statistical significance, while in the expert group the improvement was statistically significant only for the ES bypass. The NOMAT score also tended to improve in both groups, showing a statistically significant progress in the novice group for the EE bypass. The mean number of leakages after bypass execution, as well as the relative time for their resolution, also tended to slightly and progressively reduce in both groups by increasing the number of attempts, thus again underlining the potential effectiveness of the training model in refining microsurgical skills. Experienced trainees expressed a slightly better subjective evaluation of the training model than novices (Likert score of 25 vs. 24.58 out of 30, respectively).

## Limitations

Iatrotek® didactic platform and PVA hydrogel vessels, despite the above described advantages, are more expensive than other models presented in our discussion. On the other hand this bypass simulator could be used not only by neurosurgeons but also by other physicians (e.g., vascular, plastic surgeons, etc.). In this way, by purchasing only one simulator, surgeons from different specialties could improve their microvascular skills. As a consequence, PVA hydrogel vessels could be purchased in large amounts and at a more convenient price from the supplier. However, as above mentioned, PVA hydrogel vessels are more expensive than placental ones. On the other hand, PVA hydrogel vessels have no storage issues and no costs of conservation. In addition, they are ready to use and realistic because of their technique of production and mechanical properties. Hence, according to our analysis, this bypass simulator could be practical and cost-effective when compared with the alternative solutions on the market.

## Conclusions

In conclusion, our proposed bypass training model may represent a simplified, ready-to-use, reusable, ergonomic and efficient system to improve eye-hand coordination and dexterity in performing microanastomosis.

## Data Availability

The original contributions presented in the study are included in the article/Supplementary Material, further inquiries can be directed to the corresponding author/s.
